# Dengue Myocarditis: A Case Report and Major Review

**DOI:** 10.5334/gh.1254

**Published:** 2023-08-04

**Authors:** Roberto Cristodulo, Gracia Luoma-Overstreet, Fernando Leite, Manuel Vaca, Michelle Navia, Gustavo Durán, Fernando Molina, Bozorg Zonneveld, Sergio Víctor Perrone, Alejandro Barbagelata, Edgardo Kaplinsky

**Affiliations:** 1 Clínica de las Américas, Santa Cruz, Bolivia; 2 Virginia Commonwealth University School of Medicine, Richmond, VA, US; 3 Catholic University of Argentina, Buenos Aires, Argentina; 4 Duke University School of Medicine, Durham, NC, US; 5 Hospital Municipal de Badalona, Barcelona University, Barcelona, Spain

**Keywords:** Acute myocarditis, dengue, expanded dengue syndrome

## Abstract

Dengue is a viral disease transmitted by the bite of a female arthropod, prevalent primarily in tropical and subtropical regions. Its manifestations include asymptomatic infections, dengue fever, and a severe form called *hemorrhagic dengue* or *dengue shock syndrome*. Atypical manifestations can also occur, called *expanded dengue syndrome*.

We describe the case of a 43-year-old man with an unusual presentation of dengue, demonstrating a workup suggestive of myocardial and pericardial damage. Symptoms and markers indicative of cardiac compromise improved after five days on anti-inflammatory treatment.

Dengue myocarditis is considered an uncommon complication of dengue, although its reported incidence is likely an underestimation. In general, most cases of dengue myocarditis are self-limited, with only a minority at risk of progressing to heart failure. In order to improve recognition and prevent progression, healthcare providers should maintain a high degree of suspicion regarding potential cardiac complications in patients with dengue.

## Introduction

Dengue is an infection transmitted by the bite of a female arthropod, particularly the *Aedes aegypti* or *Aedes albopictus*, which has been infected by one of the four dengue virus (DENV) serotypes: DENV-1, DENV-2, DENV-3, or DENV-4 [1]. An additional fifth serotype of the virus has recently been described [2]. This disease is endemic to tropical and subtropical regions around the world, especially in urban and suburban regions, and represents an important public health problem [3].

When the *Aedes aegypti* mosquito bites a person infected by DENV, the virus replicates in the intestines of the mosquito before disseminating to its secondary tissues, including the salivary glands. The time between entry of the virus and transmission to a new host is called *extrinsic incubation*, and when the temperature is between 25°C and 28°C, this period lasts between eight to twelve days [4]. The Aedes mosquito has adapted to be able to reproduce in manmade structures including buckets, clay pots, discarded containers, used tires, stormwater drains, and more; this has rendered dengue an insidious disease in densely populated urban centers. *Aedes aegypti* mosquitos feed during the day, predominantly in the morning and evening. The female mosquito feeds frequently between spawning periods, which results in large groups of infected individuals. Once the eggs are laid, they are viable for months in dry conditions and hatch after contact with water.

The incidence of dengue has increased significantly in the last several decades; current estimates predict that about half of the world’s population is at risk of the infection. According to the World Health Organization (WHO) there are between 100 and 400 million new infections every year, although more than 80% of these are mild or asymptomatic [2]. In general, most infections follow a course similar to that of the common cold, but occasionally they can progress to serious complications and even fatal outcomes [3].

### Case report

A 43-year-old man was admitted to the Clínica de las Américas (CDLA) in Santa Cruz de la Sierra, Bolivia, presenting with moderate-intensity, continuous crushing chest pain radiating to the lower jaw and the left shoulder. The pain had started two days prior and had progressively increased in intensity.

Five days prior to his hospitalization, the patient had presented to the emergency department for fever, headache, retro-orbital pain, general body aches, and joint pain. Due to the high degree of suspicion and multiple cases of dengue in the community, a dengue test (ELISA IgM) was performed, yielding a positive result, which was subsequently confirmed through reverse transcriptase polymerase chain reaction (RT-PCR). The patient was hemodynamically stable with no signs of heart failure, exhibiting no murmurs or rubs on auscultation, and a chest-radiography performed at that time showed normal results.

**Figure 1:**
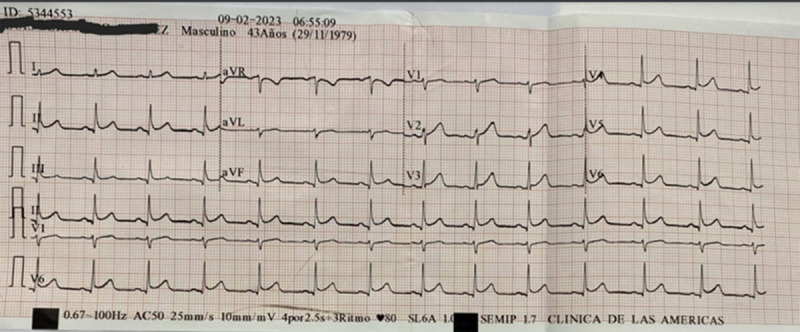
Electrocardiogram with ST segment elevation.

The patient’s electrocardiogram (EKG) revealed sinus rhythm with a heart rate of 95 beats per minute. Additionally, an ST segment elevation of 2 mm was observed in leads II, III, aVF, and V4 to V6 (Figure 1). This was interpreted as acute coronary syndrome with ST-segment elevation, so blood tests (including cardiac biomarkers) were requested and the myocardial infarction protocol (primary angioplasty) was activated. However, a coronary angiography revealed no significant lesions.

**Figure 2:**
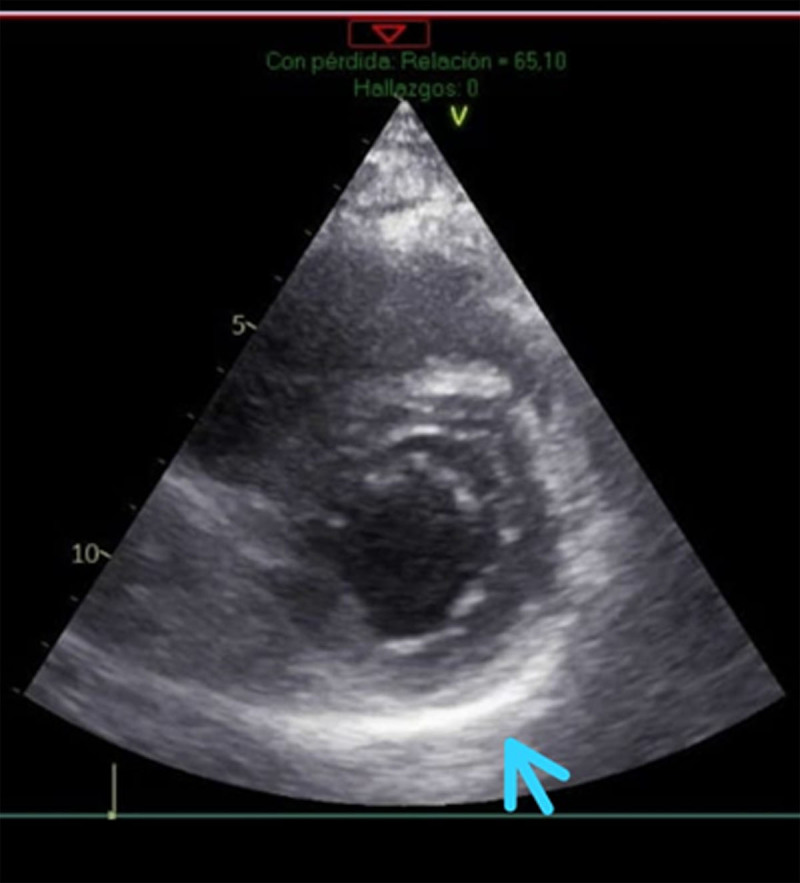
Echocardiogram of the short axis with thickening of the parietal pericardium.

Simultaneously, a transthoracic echocardiogram was performed, revealing normal systolic function in both ventricles. The left ventricular ejection fraction (LVEF), assessed using Simpson’s method, was 66%, and the diastolic function was preserved. Longitudinal deformation was –19.8% with a minor decrease in the deformation affecting the basal and medial inferoseptal segment. Finally, the parietal pericardium was found enlarged and diffusely hyperechogenic, suggesting an inflammatory process at that level (Figure 2).

Complete blood count and basic metabolic panel lab results all yielded normal results, with hemoglobin of 12.6 g/dl, hematocrit of 39.2%, leukocyte count of 8700 cells/μl with normal differential and a platelet count of 263,000/μl. Creatinine, sodium and potassium levels were 0.7 mg/dl, 142 mEq/L, and 4.6 mEq/L, respectively. Cardiac markers were slightly elevated, with a NT-proB-type Natriuretic Peptide (NT-proBNP) level of 678 pg/mL (normal value <125 pg/ml), a troponin T level of 154 ng/L (normal value <14), but with a normal creatine phosphokinase (CPK) value of 88 U/L (normal value <308 U/L).

**Figure 3:**
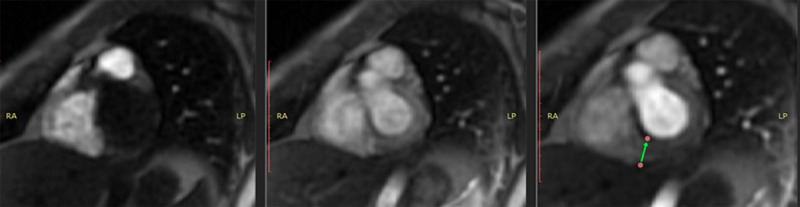
Cardiac MRI showing hyperemia in the basal portion.

Cardiac magnetic resonance imaging (MRI) with gadolinium revealed normal-sized cardiac chambers, preserved right ventricular function, and a LVEF of 52% (lower than the normal range) in a context of mild hypokinesia of the inferior wall of the left ventricle (basal and medial segments) (Figure 3).

**Figure 4:**
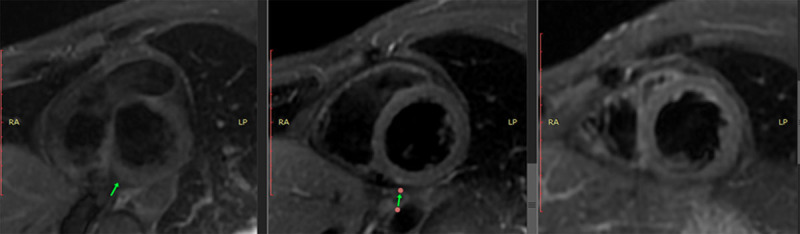
Cardiac MRI with triple IR sequencing showing hyperintense signal, compatible with myocardial edema.

**Figure 5:**
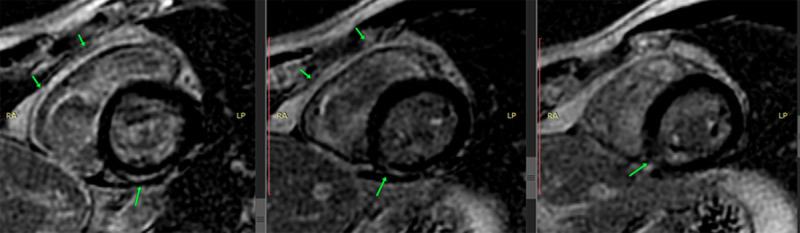
Cardiac MRI with late gadolinium enhancement in the inferoseptal, inferior, and inferomedial segments.

The presence of myocardial edema was also observed in the same inferior wall with inferoseptal extension (Figure 4). Late enhancement sequences revealed gadolinium uptake in the basal, medial, and inferior basal inferoseptal segments of the left ventricle, and the pericardium in its basal region (Figure 5). All of these findings are consistent with acute myocarditis.

The differential diagnosis also included a number of pathologies characterized by chest pain, such as acute coronary syndromes, aortic dissection, pulmonary embolism, pneumonia, pneumonitis, tuberculosis, viral myocarditis, musculoskeletal disorders, gastric ulcer, gastroesophageal reflux disease, pneumothorax, herpes zoster, depression, and panic disorder.

Considering the exclusion of acute myocardial infarction through normal coronary angiography, the clinical findings, elevated cardiac biomarkers, positive specific serology, suggestive cardiac images, and the epidemiological context, a dengue myocarditis diagnosis was suggested.

The patient was treated with nonsteroidal anti-inflammatory drugs (NSAIDs), acetylsalicylic acid, and colchicine, resulting in clinical improvement of pain and reduction in cardiac biomarkers levels. During the first 24 hours of admission, the patient had persistent hiccups, which were effectively managed with intravenous metoclopramide. Discharge was effective on the third day of admission with prescriptions for acetylsalicylic acid (750 mg every eight hours for one week) and colchicine (0.5 mg daily for two weeks).

## Discussion

### Dengue epidemiology

Dengue is a global health problem that affects more than 40% of the population in endemic regions. It is responsible for a high morbidity and mortality rate in these areas. The oldest recorded case of dengue originates from China and dates back to the Jin dynasty (265 to 420 AD). During the Neolithic period, a significant shift in lifestyle occurred as human beings transitioned from a nomadic to a sedentary way of life. With the arrival of agriculture and farming, the development of stable communities, and the emergence of primitive structures, an ideal setup for epidemics was created [7].

The abandonment of the nomadic life allowed establishing a neighborly relationship with mosquitoes, fleas, lice and/or Triatoma that serve as insect vectors of many diseases. Within these sedentary societies, pathogens acquired an endless supply of hosts and evolved to become less virulent but more infectious, ensuring the preservation of their hosts [7].

Beginning in the 18th century and especially during the industrial revolution, the flows and transport of people increased notably, first with the railway and later with the arrival of the automobile, which favored the spread of the disease. For this reason, in the early 1870s, different countries around the world began to face serious epidemics of dengue, despite the fact that the disease was only endemic in some regions of Africa, the Americas, the Mediterranean, Southeast Asia, and the Western Pacific (Asia alone had about 70% of the cases worldwide) [8].

Reaching our contemporary times, 2019 saw the largest number of dengue cases worldwide. In this context, all regions of the world were affected, and, for the first time, cases were reported in Afghanistan. In the United States alone, 3.1 million cases were documented (25,000 of them classified as serious) while the number of cases in Asia also increased significantly, such as in Bangladesh (101,000), Malaysia (131,000), Philippines (420,000), and Vietnam (320,000) [9].

During 2020 and 2021, a notable increase in dengue cases was also reported in many other countries including Brazil, Ecuador, India, Indonesia, Cook Islands, Maldives, Mauritania, Mayotte (French archipelago), Nepal, Singapore, Sri Lanka, Sudan, Thailand, East Timor, Yemen, Colombia, Fiji, Kenya, Paraguay, Peru, and Reunion Islands [10,11].

### Clinical presentation of dengue

Dengue is responsible for a wide spectrum of clinical presentations, ranging from an asymptomatic infection (where the patient may be unaware of the infection), to symptoms similar to the flu, or, more infrequently, some severe cases causing bleeding, systemic compromise, organ failure, and extravasation of plasma. Severe dengue can be fatal if not properly treated, as first reported in the 1950s during an epidemic in the Philippines and Thailand [10]. Nowadays, its presence affects most countries in Asia and Latin America, and it has become one of the leading causes of hospitalization and death in these regions [11].

It has been estimated that there are some 390 million global dengue infections every year (95% confidence interval 284 to 528 million). Around 96 million (67 to 136 million) of these cases have clinical manifestations, with varying levels of severity [12]. One study estimates that 3.97 billion people worldwide are at risk of dengue infection due to epidemiological risk factors. Of these, 824 million live in urban and 763 million in peri-urban areas [13]. The number of dengue cases notified to the WHO has increased eightfold in the last 20 years, rising from 505,430 cases in 2000 to over than 2.4 million in 2010, and reaching 5.2 million in 2019 [10].

Most dengue infections are asymptomatic or minimally symptomatic. Some patients may also present with high fever, intense pain behind the eyes, headaches, muscle pain, joint pain, nausea, vomiting, extreme fatigue, itching and/or bleeding from the nose or gums, and appearance of spots on the skin, among other symptoms. Cases of intense cough have been described in both children and adults. However, dengue can worsen and generate the well-known *dengue hemorrhagic fever*, although even in this context, fatal cases are rare [14]. The clinical appearance of the disease usually occurs following an incubation period of four to ten days after being bitten by an infected mosquito, and symptoms may last from two to seven days [10].

**Figure 6:**
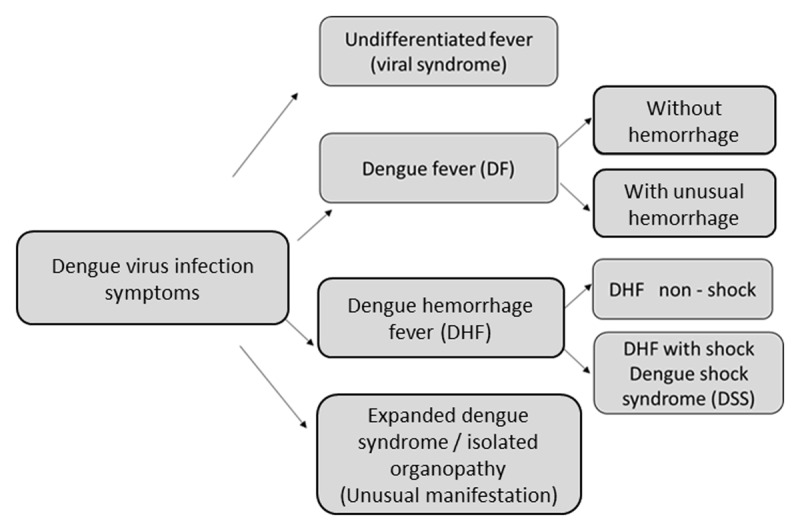
Dengue classification according to World Health Organization (WHO).

The WHO classified symptoms from dengue into the following categories (Figure 6):

**1)** *Non-classical dengue fever* or *undifferentiated fever*: simple febrile processes (indistinguishable from other viral infections), mainly in individuals who have been infected with dengue virus for the first time.
**2)** *Dengue fever without bleeding*: acute febrile illness with severe headache, myalgias, arthralgias, skin rash and bone pain (*breakbone fever*); leukopenia and thrombocytopenia may also be present.
**3)** *Dengue fever with bleeding*: clinical presentation of classical dengue accompanied by hemorrhages. Typically, the bleeding manifestations are mild and include a positive tourniquet test, petechiae, or skin bruising, epistaxis, gingival bleeding, and microscopic hematuria. Some severe forms may include vaginal bleeding, hematemesis, melena, and intracranial hemorrhage.
  **3.1)** *Dengue hemorrhagic fever (non-shock)*, defined by the following four WHO criteria: fever or recent history of acute fever lasting two to seven days; hemorrhagic manifestations (previously described); thrombocytopenia with platelet count <100,000/mm^3^; and evidence of increased vascular permeability. Plasma leakage, typically observed after the febrile phase (third to sixth day), is the key characteristic and the critical phase of the disease, and is a consequence of immune mediators rather than capillary damage. Plasma leakage includes an elevated hematocrit ≥20% above the population mean hematocrit for age and sex, a decline in hematocrit after volume-replacement treatment of ≥20% of the baseline hematocrit, presence of pleural effusion or ascites detected by radiography or other imaging method, hypoproteinemia (<6g/dL), or hypoalbuminemia (<3.5 g/dL), as determined by laboratory tests. Bleeding is not necessary for the diagnosis of dengue hemorrhagic fever when evidence of plasma leakage is detected. However, the name *hemorrhagic dengue* is maintained due to the tendency towards hemorrhage in these cases.
  **3.2)** *Dengue shock syndrome*: typical dengue hemorrhagic fever accompanied by circulatory failure (hypovolemic shock because of plasma leakage). Patients with severe leakage may develop shock with the consequent progressive organ failure, metabolic acidosis, disseminated intravascular coagulation, and eventually, hemorrhage.
**4)** *Expanded dengue syndrome/isolated organopathy*: dengue patients can occasionally develop unusual clinical manifestations as a consequence of viral invasion and direct toxicity of the liver (elevation of liver transaminases, acute liver failure), kidneys (acute renal failure, proteinuria, glomerulonephritis, hemolytic uremic syndrome, etc.), bone marrow (thrombocytopenia, lymphocytosis, pancytopenia, aplastic anemia, disseminated intravascular coagulopathy, etc.), brain (encephalopathy, encephalitis, acute disseminated encephalomyelitis, optic neuritis, etc.), or heart (arrhythmias, pericarditis and myocarditis, heart blocks, etc.) [2,5,6].

Several signs and symptoms have been identified as accurate predictors of poor prognosis, classified as warning signs. These signs are: intense and sustained abdominal pain; persistent vomiting; serous effusion (in peritoneum, pleura or pericardium); postural hypotension or syncope; mucosal bleeding; change in the patient’s mental state such as drowsiness or irritability; hepatomegaly (>2 cm); and sudden increase in hematocrit concomitant with rapid decrease in platelet count (late sign). The secondary classification of dengue, with or without alarm symptoms, was developed to assist health professionals in selecting patients for admission to the hospital and close monitoring, in order to reduce the risk of developing a more serious presentation of dengue [2].

### Cardiac manifestations

One of the first descriptions of myocarditis related to arbovirus infections (dengue or chikungunya fever), including clinical characteristics and possible sequelae, was made by Obevesekere and Hermon in 1972 in Sri Lanka [15]. One year later, also in Sri Lanka, Nagaranam, Siripala, and Nandani de Silva reported two new cases of arbovirus-related myocarditis [16]. Epidemiological data from India suggest that one in 206 dengue patients have cardiac symptoms attributed to myocarditis. Conversely, Wali et al. reported that a large number of patients with DHF present left ventricular hypokinesia and LVEF <40% [17,18].

**Figure 7:**
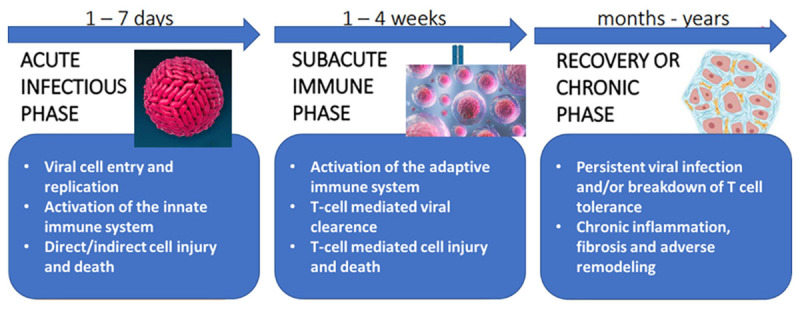
Summary of the cellular and molecular pathology of viral and autoimmune myocarditis.

A study of 1,782 patients diagnosed with dengue in China found that 201 of these patients experienced myocarditis, and 11.8% of these patients with myocarditis required hospitalization [19]. On the other hand, histological evidence of myocarditis was revealed in an autopsy study of five patients who died from other serious complications of dengue but were never diagnosed with myocarditis (subclinical myocarditis) [20]. In dengue myocarditis, the inflammatory process can affect the myocytes, the vascular structures, the conduction system, the autonomic nerves, and the interstitium, and it is not infrequent that the pericardium is affected by contiguity [19]. Myocardial damage is likely the combined effect of a direct viral attack and an added immune-mediated lesion. Both the viral genetic material and the viral proteins can directly promote myocyte apoptosis, while the immunological injury is generated by the leukocyte response and the inflammatory cascade of cytokines. In addition, vascular endothelial injury caused by the virus or by the added immune reaction also indirectly contributes to myocardial injury [21] (Figure 7).

The clinical manifestations of dengue myocarditis range from silent forms to symptoms of chest pain, dyspnea, heart failure, pulmonary edema, or cardiogenic shock. Dengue myocarditis can even imitate acute myocardial infarction [21,24], as in our case. Arrhythmias including sinus tachycardia, ventricular arrhythmias, supraventricular arrhythmias such as atrial fibrillation, and/or varying degrees of atrioventricular block can be detected [19,22,23].

The first case of sinus node dysfunction related to dengue infection was reported in a three-year-old boy who developed hypotension and bradycardia after recovering from dengue hemorrhagic fever [25]. Agudelo-Salas et al. reported that in patients without a myocarditis diagnosis, a significant presence of symptoms may suggest its presence, including tachycardia (23.1%), bradycardia (15.4%), and hypotension (12.8%) [26]. As previously stated, the cardiac effects of dengue virus extend beyond myocarditis, and can affect the conduction system, including nonspecific ST-segment alterations and low QRS complex voltage, in addition to the arrhythmias previously described. All these manifestations can either be asymptomatic or may cause severe symptoms [27].

### Establishing a diagnosis

When there is suspicion of a dengue infection in a patient with supportive epidemiologic factors, it is essential to confirm the diagnosis through serological tests. In this context, blood samples obtained in the first week of illness should be analyzed with RT-PCR. This is because the virus can only be isolated in blood during this period (specialized equipment and trained staff are required). Dengue virus can also be detected through commercially-available easy rapid diagnostic tests that are designed to identify a specific viral protein produced by the virus, known as the non-structural protein NS1. This protein is secreted into the blood during the acute phase of dengue infection, typically within the first seven days. A positive result of the NS1 test indicates a dengue infection, although it does not provide information about the specific serotype. The results of these tests can be obtained within approximately 20 minutes. Complex serological methods (such as ELISA), can be employed to confirm the presence of recent or past dengue infection. These methods detect specific antibodies against the dengue virus. IgM antibodies can be detected from approximately the first week of infection and remain detectable for about three months, indicating a recent dengue infection. IgG antibodies take longer to develop, but remain in the system for years. Their presence suggests a previous infection [10].

The diagnosis of myocarditis, an inflammatory condition of the myocardium, remains a challenge due to its heterogeneous clinical presentation and the wide spectrum of underlying etiologies [28]. It should be remembered that while myocarditis is commonly caused by viral infections and other infectious organisms, it may also be less frequently caused by systemic diseases, drugs, or toxins [29]. Although mild cases usually resolve spontaneously, myocarditis can lead to the development of dilated cardiomyopathy, present as heart failure, or sudden cardiac death [30]. Echocardiography usually reveals structural and functional abnormalities such as dilation of the cardiac chambers, systolic and diastolic dysfunction, pericardial effusion, and valvular insufficiencies.

In addition, cardiac biomarkers, including troponins and NT-proBNP, are often elevated [31]. In recent years, cardiac MRIs have become a useful non-invasive method due to their unique ability to characterize tissue in a multiparametric manner, in addition to providing prognostic information. MRIs are not only capable of detecting the same abnormalities as an echocardiography, but they also provide additional information about the presence of inflammation and myocardial edema [32]. The European Society of Cardiology (ESC) and the American Heart Association (AHA) consider MRIs an extremely useful tool for evaluating patients for possible myocarditis [33,34]. However, it should be noted that endomyocardial biopsy remains the gold standard for establishing a definitive diagnosis of myocarditis. Nonetheless, this method has several limitations, including the invasive nature of the test, low sensitivity, and the need for trained medical personnel to perform the procedure, which contributes to its infrequent clinical use [33,34].

### Treatment and prevention

There is no specific treatment for dengue. Management includes rest, adequate hydration, and seeking medical advice for complications [35]. Depending on the clinical manifestations and other circumstances, patients may be discharged and sent home, recommended for inpatient management, or require emergent treatment and urgent referral. To control muscle aches and fever, patients can take analgesics, but should avoid NSAIDs such as ibuprofen or aspirin due to risk of hemorrhage [36]. On the other hand, NSAIDs are considered a first-line treatment for pericarditis and pericardial damage, as benefits outweigh potential risk of bleeding.

The first vaccine against dengue was Dengvaxia® (CYD-TDV), developed by Sanofi Pasteur and approved in 2015, with commercialization initially authorized in 20 countries. In November of 2017, a retrospective study examined the serologic state of participants at the time of vaccination and their subsequent risk of experiencing severe manifestations of dengue. The analysis revealed that the subgroup of participants in that were seronegative when first vaccinated had a greater risk of developing severe dengue and requiring hospitalization compared to those who were not vaccinated. For this reason, the CYD-TDV vaccine is now intended exclusively for people aged nine to 45 residing in endemic regions who have experienced at least one prior dengue infection. Ongoing research is exploring other potential dengue vaccines [37].

Finally, several risk factors can predict the likelihood of patients developing severe desngue disease, including advanced age, arterial hypertension, and diabetes. The prevalence of myocarditis in patients with dengue is reported to range between 3 to 5% in several studies, and a small portion of those cases progress to develop decreased LVEF. The physiology of myocardial injury is still not well understood, but is believed to be related to direct invasion of the virus to the cardiac muscle and the immune response mediated by cytokines [38,39]. These cytokines play an important role in the development of myocardial injury.

Our patient improved without severe complications; however, evidence of impaired mobility, particularly in the inferior wall, was observed in the cardiac MRI. Further studies are needed to differentiate which dengue serotype is associated with higher risk of cardiac complications.

## Conclusion

Human infection by dengue virus can have several atypical presentations, one of which is acute myocarditis. Fortunately, this complication is usually self-limited, and it is rare for cases to progress to dilated cardiomyopathy, heart failure, or death. Medical personnel must maintain a high index of suspicion for patients with epidemiological risk factors and presenting with symptoms suggestive of cardiac involvement (such as chest pain, dyspnea, tachycardia, etc.). This vigilance is crucial to avoid missing a diagnosis. Considering the likelihood of underdiagnosis, it is crucial to conduct further studies and gather additional epidemiological data to identify which patients with dengue are at a higher risk of developing cardiac complications, such as myocarditis.
